# A macro to micro analysis to understand performance in 100-mile ultra-marathons worldwide

**DOI:** 10.1038/s41598-023-28398-2

**Published:** 2023-01-25

**Authors:** Mabliny Thuany, Katja Weiss, Elias Villiger, Volker Scheer, Nejmeddine Ouerghi, Thayse Natacha Gomes, Beat Knechtle

**Affiliations:** 1 Centre of Research, Education, Innovation and Intervention in Sport (CIFI2D), Faculty of Sport, University of Porto, Porto, Portugal; 2 Medbase St. Gallen Am Vadianplatz, Vadianstrasse 26, 9001 St. Gallen, Switzerland; 3 Institute of Primary Care, University of Zurich, Zurich, Switzerland; 4 Klinik Für Innere Medizin, Kantonsspital St. Gallen, St. Gallen, Switzerland; 5 Ultra Sports Science Foundation, 109 Boulevard de L’Europe, 69310 Pierre-Benite, France; 6 University of Jendouba, High Institute of Sport and Physical Education of Kef, UR13JS01, 7100 Kef, Tunisia; 7 Faculty of Medicine of Tunis, University of Tunis El Manar, Rabta Hospital, LR99ES11, 1007 Tunis, Tunisia; 8 Post-Graduation Program of Physical Education, Department of Physical Education, Federal University of Sergipe, São Cristóvão Sergipe, 49100-000 Brazil; 9 Department of Physical Education and Sport Sciences, University of Limerick, Limerick, V94T9PX Ireland

**Keywords:** Environmental impact, Environmental social sciences, Sustainability

## Abstract

The purposes of this study were (i) to describe differences in participation in 100-mile ultra-marathons by continent; (ii) to investigate differences in performance between continents; and (iii) to identify the fastest runners by continent and country. Data from 148,169 athletes (119,408 men), aged 18–81 years, and finishers in a 100-miles ultra-marathon during 1870–2020 were investigated. Information about age, gender, origin, performance level (top three, top 10, top 100) was obtained. Kruskal–Wallis tests and linear regressions were performed. Athletes were mostly from America and Europe. A macro-analysis showed that the fastest men runners were from Africa, while the fastest women runners were from Europe and Africa. Women from Sweden, Hungary and Russia presented the best performances in the top three, top 10 and top 100. Men from Brazil, Russia and Lithuania were the fastest. The lowest performance and participation were observed for runners from Asia. In summary, in 100-miles ultra-marathon running, the majority of athletes were from America, but for both sexes and performance levels, the fastest runners were from Africa. On a country level, the fastest women were from Sweden, Hungary and Russia, while the fastest men were from Brazil, Russia and Lithuania.

## Introduction

The athletes’ performance is influenced by both individual (e.g., genetic, morphological, training) and environmental factors (e.g., coach, family, social characteristics)^1,2^. Moving beyond the athlete-centered approach, recent studies were developed to understand the role of the environment in the athlete’s performance^3–5^. The ‘birthplace effect’ has been largely studied in team sports, such as soccer^6^, ice hockey^7^, basketball^8^, volleyball^9^, and handball^10^.

Furthermore, among individual sports such as running, the interest in understanding the link between the environment and the athletes’ performance has increased in the last years^11,12^. These interests were associated with the increasing numbers of both runners and running events across the world^13^, especially after the 1970’s in North America and after the 1980’s in Europe^14^.

There is ample evidence that the fastest long-distance runners, such as marathoners, originate from the African continent, particularly from Kenya and Ethiopia^15,16^. This representation is related to a plethora of factors, which include–but are not limited to–physiological characteristics, training, lifestyle behaviors, and motivational factors^17,18^. Considering Brazil, the Southeast region as the richest region of the country is the region with the highest number of elite long-distance runners in the country^19^.

However, in the context of ultra-marathon running, little is known about where the fastest ultra-marathoners come from. One of the few studies found that Russian and Japanese were the fastest for the 100-km ultra-marathon race distance^16,20^. Similar results were found by Cejka et al.^21^, where most of the finishers in 100-km ultra-marathons were from Europe, but Japanese runners were the fastest. On the other hand, data covering 96,036 athletes (88,286 men and 7,750 women) finishing the oldest 100-km ultra-marathon in the world (‘100 km Lauf Biel’ in Switzerland), showed that Switzerland, Germany, and France were the countries with the highest number of participants throughout the history of the race^22^. These initial insights suggest that the place of the competition and the countries’ economic indicators are related to these results.

To date, most studies regarding participation and performance trends have shown an increase in the number of finishers in the last decades’^23,24^, especially in events hosted in the USA, where most of the events were taking place^24,25^. Notwithstanding the relevance of these studies, it is important to present a more generalized view by using a macro-level approach investigating the between-continents differences, followed by a micro-level analysis within-country. It has also been shown that athletes from specific countries improved their performance over years in specific races such as the ‘Spartathlon’^26^ whereas in other races such as the ‘100 km Lauf Biel’, the performance of athletes from specific countries decreased^22^. Generally, the fastest athletes were able to improve their performance across years in the world’s most famous ultra-marathon races such as the ‘Spartathlon’^26^ and the ‘Comrades’^27^.

In this sense, the purposes of this study were (i) to verify the participation of athletes by continent and country in 100-mile ultra-marathons (161-km) performed between 1870 and 2020, (ii) to compare the athletes’ performance between continents and countries, (iii) to identify the fastest athletes by country and by continent and, (iv) to investigate the trend in performance over years of the athletes from the fastest countries. Previous studies showed that North America and Europe presented higher numbers of ultra-marathon events worldwide (431 and 455, respectively)^25^ compared to Asia (139), Africa (59), Australia (35), South America (18), as well as a higher participation in 161-km ultra-marathons^28^. For another way, Russian athletes were the fastest in long-distance running races such as the ‘Comrades Marathon’^29^, and in 100-km ultra-marathon running races^20^. Based on it, we hypothesized that (i) the highest number of athletes would be found on the American continent, especially in USA and Canada, while Russian runners would be the fastest and (ii) the fastest runners would be able to improve their performance over time.

## Methods

### Ethical approval

The study was performed following the Declaration of Helsinki, and the institutional review board of St Gallen, Switzerland, approved this study (EKSG 01/06/2010). Since the study involved the analysis of publicly available data, the requirement for informed consent was waived. Participants were not identified during the data management and during all sections of the manuscript.

### Design and sample

Data used in the present study was obtained from the website of the ‘Deutsche Ultramarathon-Vereinigung’ DUV (https://statistik.d-u-v.org/geteventlist.php) and corresponded to the officially available results of the participants enrolled in 100-mile (161 km) ultra-marathon race events held during 1870–2020 for both genders. The available information included the year of the event, race distance, year of birth, gender, general ranking, country, team, mean running speed, and race time. Based on this information, we clustered the athletes by country and then by continent (e.g., Africa, America, Asia, Oceania, and Europe). Performance levels were categorized based on the general classification considering the top three, top 10 and top 100 for both genders. Exclusion criteria were: athletes aged below 18 years, athletes clustered in countries with less than 10 participations (when comparison within a continent was made), mean running speed higher than 20 km/h, and missing information about the country of origin.

### Statistical analysis

Descriptive information was presented in mean (standard deviation), minimum (min), maximum (max) values, and frequency (%). Data normality was formally tested using the Kolmogorov–Smirnov test. Based on the athletes’ performance, runners were classified considering their ranking position (top 100, top 10, and top three), for both genders. Following, athletes were clustered based on their birthplace (e.g., country and continent). To compare the performance level according to the continent, we performed the Kruskal–Wallis test, followed by the post-hoc test adjusted by the number of comparisons.

A simple linear regression was performed to verify the performance trends. Running speed (miles/h) was the outcome variable, while the time (year) was considered as a predictor. The regression was performed considering both, the total sample and the ranking position (top three, top 10, and top 100), as well as the three best countries over time for both genders. For the total sample regression analysis, we considered data from the 1970s, since a running boom in high and middle-income countries from this decade was verified ^30^. For the three best countries according to the performance level, we considered data from 2010. Data analysis was performed in the SPSS 26, and the significance level was set at 0.05.

## Results

A total of 148,169 athletes aged 18–81 years and of both genders (women = 28,761; men = 119,408), finished at least one 100-mile ultra-marathon during 1870–2020. Athletes came from 113 countries from all five continents. Most of them were from the American continent (72.7%), followed by Europe (15.9%), Asia (5.6%), Africa (3.7%), and Oceania (2.1%). Running speed distribution between athletes showed a similar pattern for all continents. Most of the runners presented mean values of 5–7 miles/h, and a small number of athletes presented running speeds higher than 10 miles/h. Mean running speed was 6.12 miles/h for the total sample (women = 5.96 miles/h; men = 6.21 miles/h).

### Participation and performance by continent—a macro-analysis

**Figure 1:**
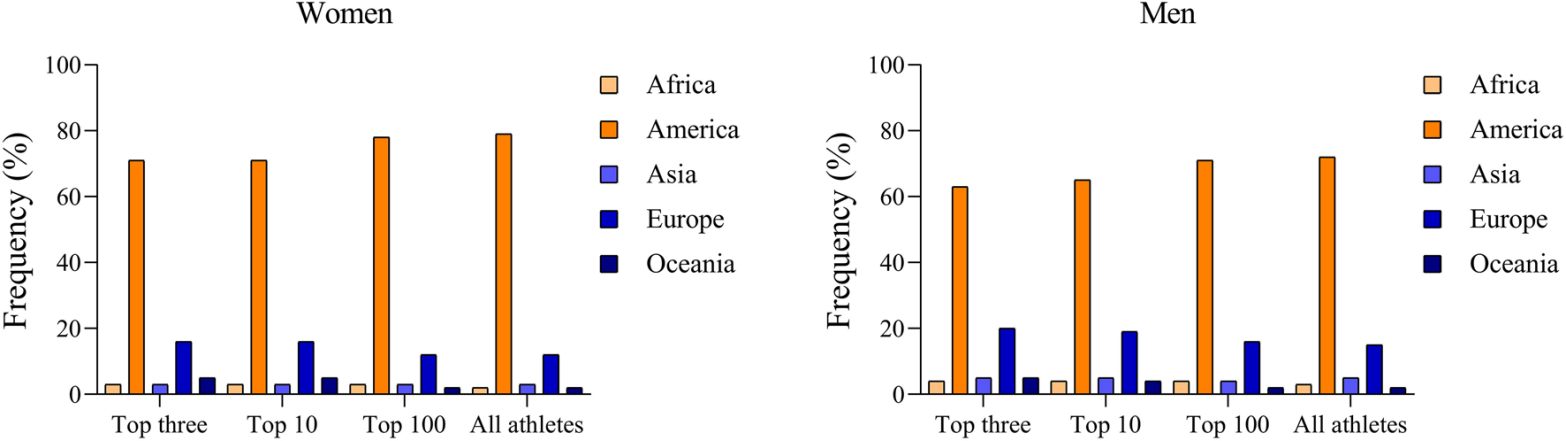
Percentage of women and men participation according to performance level.

Figure 1 presents the distribution of the athletes, according to their continent of origin into different performance groups (top three, top 10, top 100, and all athletes) for both genders. Most women in all the performance levels were from the American continent, followed by Europe. Visual differences are presented for the top three and top 10, where Oceania has a higher number of athletes compared to the African continent (e.g., top three and top 10).

Considering athletes clustering by continent, significant differences in performance were shown (H_(4)_ = 1766.22; *p* < 0.001). The descriptive analysis showed that African women achieved the highest mean running speed (6.89 ± 1.04 miles/h), followed by athletes from Europe (6.45 ± 1.38 miles/h), America (5.89 ± 1.01 miles/h), Oceania (5.85 ± 1.37 miles/h), and Asia (5.17 ± 1.49 miles/h). Significant differences were observed between most of the continents, except between America and Oceania (*p*-adjusted = 0.178). For men, for all the performance levels (top three, top 10, top 100 and all athletes), most of the athletes were from both the American and the European continent. The lowest percentages of participation were found for Oceania. Considering the performance by continent, athletes from Africa presented the highest mean values for running speed (7.42 ± 1.33 miles/h), followed by Europe (6.53 ± 1.53 miles/h), Oceania (6.39 ± 1.59 miles/h), America (6.13 ± 1.17 miles/h), and Asia (5.34 ± 1.47 miles/h). Differences in the performance were found between continents (H_(4)_ = 859.43; *p* < 0.001), with significant differences between all of them.

### Participation and performance by country—a micro-analysis

For the top three athletes, a total of 36 and 65 countries were listed for both women and men, respectively. Most of the athletes were from the USA (62.1% and 56.9% for women and men, respectively). Similarly, the highest number of athletes from the USA were observed in the top 10 (62.6% and 58.4% for women and men, respectively) and top 100 (72.4% and 66% for women and men, respectively). Therefore, within the European continent, the majority of the athletes were from Germany and the Great-Britain for all performance levels.

Table 1 presents the results for the comparison between performance levels for both genders. Significant differences were observed for both genders and all performance levels. For men athletes, the fastest runners were from the African continent. For the top three women, the fastest ones were from Europe, while for the top 10 and top 100 the fastest were Africans.

**Table 1:** Descriptive results (mean ± SD for miles/h) for performance (speed mean, mile/h) according to gender and performance level.

	Men			Women		
	Top three	Top 10	Top 100	Top three	Top 10	Top 100
Africa	9.37 (1.7)^abcd^	8.64 (1.6)^abcd^	7.45 (1.3)^abcd^	7.52 (1.7)	7.39 (1.5)^bc^	6.91 (1.0)^abc^
America	7.93 (1.5)^a^	7.28 (1.4)^a^	6.29 (1.2)^ab^	7.37 (1.7)^d^	6.80 (1.4)^a^	6.04 (1.1)^ab^
Asia	7.10 (1.8)	6.59 (1.7)	5.76 (1.5)	7.29 (2.1)^d^	6.44 (1.8)^a^	5.71 (1.5)
Europe	8.29 (1.9)^abc^	7.64 (1.8)^abc^	6.74 (1.5)^ac^	8.29 (1.8)	7.38 (1.7)^abc^	6.72 (1.4)^abcd^
Oceania	8.05 (1.7)^a^	7.27 (1.6)^a^	6.45 (1.6)^a^	7.45 (1.5)	6.71 (1.4)	5.91 (1.4)^a^

*SD* standard deviation.

^a^difference for Asia; ^b^difference for America; ^c^difference for Oceania; ^d^difference for Europe.

Table 2 presents the descriptive results for women runners, considering athletes from the fastest country in each continent and the performance level (e.g., top three, top 10 and top 100). For the African continent, the fastest women were from South Africa, in the three performance groups. For the other continents, there was no verified pattern of association between the countries and performance levels, nonetheless, the highest running speeds were observed in the European continent when compared to the other continents. Among the top three, top 10 and top 100, women from Sweden, Hungary and Russia achieved the fastest running speeds.

**Table 2:** Women descriptive results (min, mean ± SD, max) for the best country in each continent, based on performance level.

	Country	Total athletes	Speed (miles/h) (Min–Max)	Speed (miles/h) (Mean ± SD)
Top three				
Africa	South Africa	35	3.08–10.81	7.52 (1.72)
America	Canada	82	4.35–10.00	7.44 (1.23)
Asia	Philippines	13	5.14–6.36	5.71 (0.36)
Europe	Sweden	17	5.52–10.60	8.31 (1.47)
Oceania	Australia	33	4.46–10.44	7.55 (1.35)
Top 10				
Africa	South Africa	150	2.41–10.81	7.39 (1.52)
America	USA	2658	2.80–19.42	6.82 (1.50)
Asia	Japan	26	4.08–11.59	8.13 (2.02)
Europe	Hungary	11	7.59–11.12	9.07 (1.06)
Oceania	New Zealand	48	3.41–9.64	6.87 (1.55)
Top 100				
Africa	South Africa	713	2.41–10.81	6.92 (1.05)
America	El Salvador	11	5.47–8.06	6.65 (0.83)
Asia	Cyprus	67	3.38–10.37	7.21 (1.52)
Europe	Russia	55	5.19–10.29	8.05 (1.21)
Oceania	Australia	445	3.38–10.44	5.93 (1.36)

Min–Minimum value; Max–Maximum value. *SD* standard deviation.

We only considered the best country for each continent. Countries with total athletes below 10 were not considered.

Men descriptive results for athletes from the fastest countries in each continent are presented in Table 3. For the top three, the fastest athletes were from America (Brazil: 9.54 ± 1.75 miles/h). In the top 10 and top 100, athletes from Europe (e.g., Russia and Lithuania) presented the highest mean running speed compared to the other continents.

**Table 3:** Men descriptive results (min, mean ± SD, max) for the best country in each continent, based on a performance level.

	Country	Total athletes	Speed (miles/h) (Min–Max)	Speed (miles/h) (Mean ± SD)
Top three				
Africa	South Africa	411	3.14–13.47	9.35 (1.73)
America	Brazil	16	4.97–11.65	9.54 (1.75)
Asia	Taiwan	62	3.36–10.24	8.25 (1.47)
Europe	Hungary	32	5.73–11.95	9.48 (1.60)
Oceania	Australia	374	4.19–13.21	8.07 (1.72)
Top 10				
Africa	South Africa	1166	2.52–13.47	8.63 (1.67)
America	Brazil	28	4.97–11.65	8.77 (1.91)
Asia	Taiwan	214	3.36–10.49	7.97 (1.22)
Europe	Russia	100	5.03–14.03	9.36 (1.51)
Oceania	New Zealand	239	3.38–12.39	7.31 (1.60)
Top 100				
Africa	Zimbabwe	12	4.61–11.88	8.01 (2.60)
America	Brazil	64	3.43–11.65	7.61 (2.00)
Asia	Israel	23	4.01–10.49	7.50 (1.78)
Europe	Lithuania	13	4.31–12.53	8.41 (2.05)
Oceania	Australia	1850	3.42–1.21	6.51 (1.60)

Min–Minimum value; Max–Maximum value. *SD* standard deviation.

We only considered the best country for each continent. Countries with total athletes below 10 were not considered.

**Figure 2:**
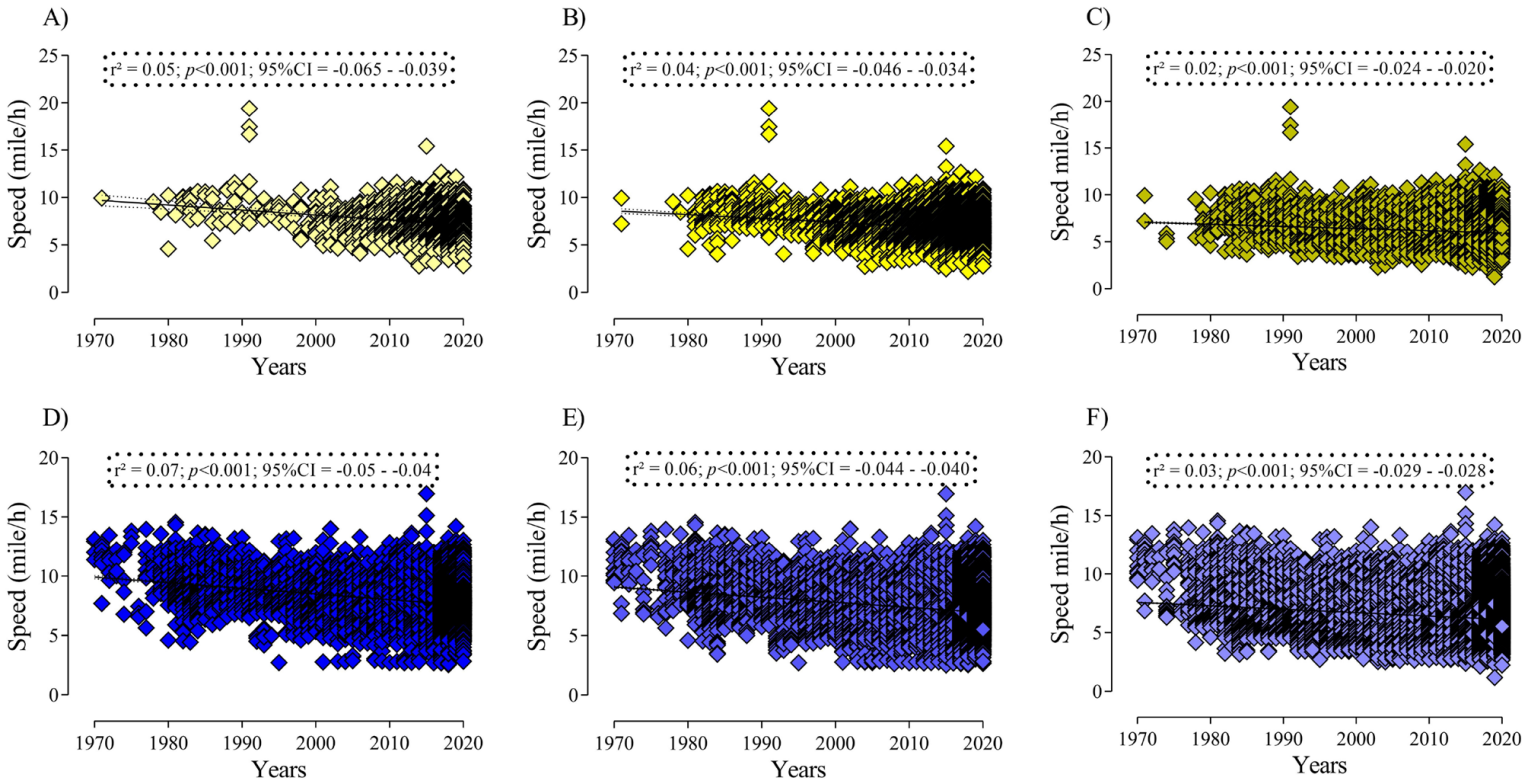
Linear regression results, considering all sample (**A**) Women top three; (**B**) Women top 10; (**C**) Women top 100; (**D**) Men top three; (**E**) Men top 10; (**F**) Men Top 100).

Figure 2 presents the performance trend results for both genders considering all the sample. For all performance groups and both genders, performance decreased over time (*p* < 0.001).

**Figure 3:**
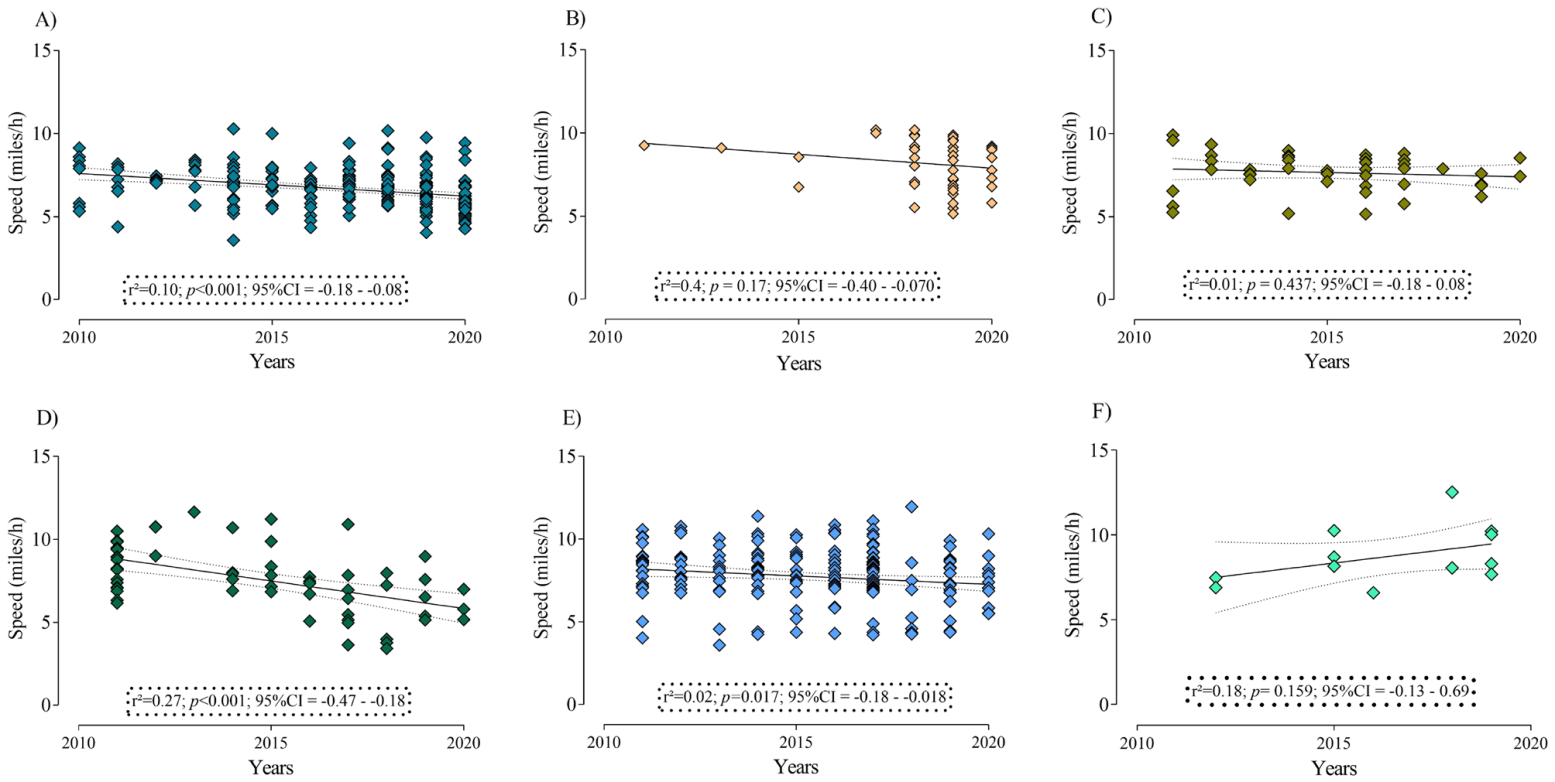
(**A**) Sweden women top three; (**B**) Hungary women top 10; (**C**) Russian women top 100; (**D**) Brazilian men top 3; (**E**) Russian men top 10; (**F**) Lithuanian men top 100.

Figure 3 presents the linear regression results for performance across time considering athletes from the best countries in the top three, top 10, and top 100, respectively. For women, runners from Russia, Hungary, and Finland were the fastest, while runners from Brazil, Russia, and Lithuania were the fastest among men. The time frame considered was the last 10 years (from 2010), in which a significant performance decline was shown for women from Sweden (*r*^2^ = 0.10; *p* < 0.001; 95%CI =  − 0.18– − 0.08). Based on the *r*^2^ values, the magnitude of the performance decline was about 0.10miles/h across the years. For men, performance decline was showed for Brazilian (*r*^2^ = 0.27; *p* < 0.001; 95%CI =  − 0.47 – − 0.18) and Russian runners (*r*^2^ = 0.02; *p* = 0.017; 95%CI =  − 0.18 – − 0.018), in which a decline of 0.27 miles/h and 0.02 miles/h was showed across the years, respectively. Athletes from Lithuania presented a visual increase in running speed over the last 10 years, however, a non-significant effect was shown.

## Discussion

The purpose of this study was to identify where the fastest runners in 100 miles ultra-marathons come from, considering the performance level. The main results showed that (i) for both genders and all performance levels, most of the athletes were from the American and European continents; (ii) a macro-analysis showed that the fastest men were from Africa, while the fastest women were from Europe and Africa; (iii) women from Sweden, Hungary and Russia presented the best performance in top three, top 10 and top 100; (iv) men runners from Brazil, Russia and Lithuania were the fastest in top three, top 10 and top 100, respectively; and (v) the lowest performance and participation were achieved by athletes from Asia.

### Participation and performance by continent—a macro-analysis

The first important finding was that most of the finishers were from America, but African runners were the fastest when analysis was performed by continent. These findings confirm our hypothesis. For both genders, the highest runner’s frequency was from the American continent, especially from the USA. Similar results were reported by Hoffman^23^ in 100 miles (161 km) ultra-marathon running competition in North America. The authors showed that from 1977 to 2008, the number of annual finish rates increased, but no improvements in performance were verified^23^.

The results of the present study can be related to the American cultural and fitness revolutions of the 1960s and 1970s, which included a ‘running boom’^13^. Specifically for the ultra-marathon races, a historical perspective ˗ the USA was one of the main ultra-marathon birthplaces around the world^31^—can influence the highest number of race events and athletes from these countries. In addition, the increase in participation among women and older athletes can be associated with this result^32^. A previous report covering approximately 107 million race results from 1986 to 2018 showed that the USA was the country with the highest number of runners, but with the slowest athletes^33^. Accordingly, the highest proportions of women participants were from USA and Canada, while Switzerland and Italy were the countries with the lowest women participation^33^.

The highest participation of these countries can be related to the highest number of ultra-marathon events performed in these countries^25^. Regarding continents, 443 ultra-marathon events were developed in America, where 431 are situated in North America. Following, Europe hosted 421 events. For data used in this study, more than half of the race events were performed in EUA (58.5%), with about 20% performed in Great Britain (5.6%), Australia (4.8%), South Africa (4.7%), Canada (4.6%), and Germany (3.4%). Besides the higher events performed in-locus, the athletes’ socioeconomic characteristics can also be related to running participation^34,35^. Athletes from a high-income country can present a better contextual indicator for traveling and participating in remote events^36^. In another way, the lowest performances showed for athletes from the USA can be related to changes in running motivation across the years. As shown in studies that include short to long-distance events, the psychological, social, and physical are the main reasons for running^37–39^, especially in non-professional athletes.

The macro-analysis has shown that the African continent presented the best mean values for running speed. This is an interesting finding, considering that African athletes are the strongest in long-distance running such as half-marathon and marathon^15,40^. However, these runners are from Kenya and Ethiopia, different from the present results, where most of them are from South Africa. These results are similar to findings in a previous report covering 85% of ultra-running events worldwide during 1996–2018, including trail runs, mountain runs and road runs. South Africa was the country with the fastest athletes, with a running pace of 10:36 min/mile, followed by Sweden (11:56 min/mile), Germany (12:01 min/mile), Netherlands (12:41 min/mile), and Great-Britain (12:44 min/mile), while the slowest were from Argentina (15:20 min/mile), Mexico (15:30 min/mile) and Malaysia (15:55 min/mile). These results were also associated with findings that countries from Asia presented the poorest performance^36^.

### Participation and performance by country—a micro-analysis

The micro-analysis showed that athletes from Sweden, Hungary, and Russia presented the best performance in the top three, top 10, and top 100 for women, and those from Brazil, Russia, and Lithuania were the fastest in the top three, top 10, and top 100 for men. We hypothesized that the fastest runners would originate from Russia, but the results partially disagree. These differences can be related to the methodological approach for the present study where we present the data for performance level (i.e., top three, top 10, top 100). For example, Nikolaidis et al.^16^, in a study including athletes ranked in World Athletics (i.e., IAAF) during 1999–2015, showed that among women, athletes from Russia were faster than athletes from France and Germany in ultra-marathon events. Similar results were shown in athletes who finished a 100-km ultra-marathon between 1959 and 2016^20^, when considering the top 10 by nationality, runners from Russia and Hungary were the fastest.

Men from Brazil in the top three are untypical considering previous studies^16,20,41^. Notwithstanding, regarding the increase in runner’s participants and race events across the country^42,43^, few studies were developed to understand the participation and performance in ultra-running events^44,45^. The country characteristics, which include variations in weather, altimetry, nutritional habits, cultural aspects, and lifestyle among the regions, should be investigated in future studies to understand the association with performance in ultramarathon events.

Considering both, the total sample and the fastest countries, performance decreased over time for both genders and performance levels. These results are similar to previous findings^23,32^. These results can be linked to the changes in the runner’s profile (e.g., intrapersonal motivation, training background, previous experience)^46–48^, and event characteristics (weather, altimetry). Differently, athletes from Lithuania showed an increase in performance over the last few years. This increase was not statistically significant, however, factors that explain these results can be related to the low number of athletes over the years, which can bias the results. The generalization of the present findings need to be considered carefully. In another way, the decrease in performance in other countries is according to previous findings, showing that countries have slowed down over the last 10 years and that those with have slowed down most are among the slowest in the rankings^36^.

More and more is known about the factors that predispose to achieve outstanding results in ultramarathon running, but without pointing to the most important ones^49^. Gajda et al. considered the success in ultra-marathons as a complex multifactorial cause and called them the “mosaic theory”. Among the factors that guarantee success they mention genetic factors such as the presence of haplogroup H mtDNA (subgroup HV0a1, belonging to the HV cluster), characterizing athletes with the greatest endurance^49^. Normal resistance to pain is also important^50^. However, none of these factors isolated guarantees success for an individual athlete or a particular nation in ultra-marathons. Additional investigations considering the environment (natural, built, and social) are necessary.

### Limitations and strengths

Limitations of the study are related to the nature of the data used. The accuracy of the data (e.g., the race distance in each event, the accuracy of the information in the first years, and the information about the birthplace of the athletes), as well as the missing data in specific time frames, and sample size variability between countries need to be considered. These limitations are challenged to be solved. To reduce the bias, the countries with a total number of athletes below 10, as well as the regression analysis with data before the 1970s were not considered. In addition, information about individual (e.g., training volume and intensity, running experience, running strategies) and contextual factors (i.e., number of competitions, economical support, cultural aspects and race course, different elevation changes, weather) are unavailable. Individual and contextual characteristics are helpful to deeply understand runners’ profile, as well as to deeply understand the impact of hosting events’ effect, and the characteristics of the race course for runners’ performance. The role of the individual characteristics for ultramarathon performance was previously investigated, however, little information is available about the role of social, economic, cultural, and geographical characteristics to increase the participation, as well as the role of the participation in performance outcomes. Future studies need to consider data triangulation, including the place in which competitions are performed, the participation and the performance outcomes, adopting different strategies regarding the performance level and sample size required within countries. Finally, we did not control for the migration or multiple events participation across the years, that is, an athlete can have moved to represent another country than his/her home country or take part in more than one event over the years. In another way, we presented a detailed analysis, considering both macro-and micro-level approaches. Since the highest number of ultramarathon events are performed in North America and Europe, even though considering mean values the fastest are from Africa and Europe, the practical application of the present study includes supports local sport police programs to increase the availability of events in countries which they are underrepresented. Athletes living in countries with a higher number of events present a higher participation rate, since the costs (travel, host) are lower^51^. Additionally, being familiar with local characteristics (language, cultural habits, and weather) is associated with performance improvement^51^.

## Conclusion

For the 100-miles ultra-marathons, most of the athletes were from the American and European continent, despite the fastest being from Africa. A micro-analysis showed that European countries’ (Sweden, Hungary, and Russia) were the best for women, while for men, Brazil, Russia and Lithuania were the fastest in the top three, top 10, and top 100. Regarding the best countries, a decrease in performance was shown over time, except for athletes from Lithuania. These results can be used to public policies to provide the highest number of race events among countries, especially those in Asia and Oceania, which showed the lowest engagement.

## Data Availability

The datasets generated during and/or analyzed during the current study are available from the corresponding author on reasonable request.
